# The Influence of Thickness on the Mechanical Behaviors of 3D Printing Resins for Orthodontic Retainers

**DOI:** 10.1155/2024/7398478

**Published:** 2024-06-25

**Authors:** Theerasak Nakornnoi, Patamaporn Bunjerdjin, Peerapong Santiwong, Kawin Sipiyaruk, Siew Peng Neoh, Rochaya Chintavalakorn

**Affiliations:** Department of Orthodontics Faculty of Dentistry Mahidol University, Bangkok, Thailand

## Abstract

This study aimed to evaluate the mechanical behaviors of thermoformed and 3D-printed retainers with different thicknesses. Thermoformed retainers (Duran) and 3D-printed retainers (Dental LT Clear V2 and NextDent Ortho Flex) were fabricated at thicknesses of 0.5, 0.75, and 1 mm. Five samples of each material were subjected to compression, tensile, and flexural testing with the universal testing machine (Instron Ltd., Buckinghamshire, England). The results revealed that the mechanical behaviors were significantly influenced by thickness in each type of material. The increased thickness tended to increase strength and modulus in all three tests. However, Dental LT Clear V2 and Duran showed that flexural strength and modulus were inversely related to thickness. The compressive test revealed significantly greater compressive resistance in 3D-printed groups, except for the NextDent Ortho Flex at 0.5 mm. The tensile test showed that Dental LT Clear V2 at all thicknesses demonstrated significantly higher tensile strength and modulus, while NextDent Ortho Flex was significantly lowest at any thickness in tensile and flexural properties. In conclusion, the thickness significantly influenced the mechanical behaviors of the 3D-printed retainers. The 0.75 mm thickness of Dental LT Clear V2 could be considered as an alternative to fabricated retainers due to its similar mechanical properties compared with the thermoformed material.

## 1. Introduction

The utilization of 3D printing technology is becoming increasingly popular in modern dentistry. Direct 3D printing appliances can be fabricated in fewer steps, offering a number of advantages, including shorter supply chains, significantly reduced fabrication duration, and lower production costs. Moreover, it is more practical for clinical use than conventional vacuum-formed devices in terms of geometrical accuracy and mechanical properties. This includes higher precision, greater load-bearing capacity, higher yield strength, and lower displacement with reversible deformation [1, 2]. Additionally, available evidence indicated that 3D-printed occlusal devices provide better dimensional stability and higher compressive resistance [1, 3]. Furthermore, it demonstrated adequate mechanical strength to maintain clinical performance under external loads [2]. Consequently, the adoption of digital technology has encouraged dental professionals and organizations to explore new materials for 3D printing. However, despite the availability of various biocompatible resins for dental applications, there is currently insufficient information regarding the utilization of 3D printing materials for fabricating dental retainers.

Biocompatibility is a critical consideration in dental materials, particularly when they interact with tissues over extended periods. A comprehensive review has highlighted a range of biocompatible resins available for the fabrication of retainers [4]. Among these, Dental LT Clear V2 resin emerges as a new generation material specifically designed to enhance its longevity and resistance to discoloration over time. Additionally, NextDent Ortho Flex resin has recently been introduced, offering superior break resistance, enhanced accuracy, and increased flexibility. These materials are classified as IIa biocompatible resins, making them suitable for use on skin and mucosa surfaces for extended periods of time. However, research investigating the physical and mechanical properties of these materials used for 3D printing retainers has been limited, despite their crucial role in determining the stability and durability of orthodontic appliances [5]. Therefore, acquiring a comprehensive understanding of these properties is essential for optimizing retainer fabrication.

Orthodontic retainers are essential for maintaining the teeth in their corrected position and preventing relapse after the completion of active orthodontic treatment. The retainer should be of adequate thickness to resist the forces exerted by the occluding dentition; however, it should be thinner to reduce its fabrication cost and to maximize patient comfort. Interestingly, clear retainers with different thicknesses may have an effect on mechanical characteristics, including flexural strength and tensile strength. There has been clinical evidence that 1 mm clear retainer could achieve lower rate of fracture than those with 0.75 mm thickness [6]. An in vitro study also demonstrated that increasing the thickness of thermoplastic materials enhances their rigidity and stability [7]. However, there has not been sufficient evidence to provide insight into the effect of thickness on the mechanical behaviors of 3D-printed clear retainers. Additionally, the optimal thickness of clear retainers may vary depending on the material used. Consequently, the purpose of this study was to investigate the effect of thickness on the mechanical behaviors of 3D printing resins for orthodontic retainers.

## 2. Materials and Methods

### 2.1. Materials for Clear Retainer Fabrication

The materials used for the fabrication of retainers in this research are Duran (Scheu-Dental), Dental LT Clear V2 (Formlabs), and NextDent Ortho Flex (Vertex-Dental B.V.). The composition of each material is listed in Table 1.

### 2.2. Sample Size Calculation

The sample size was determined using an error rate of 0.05 and a study power of 0.8. According to Ryu et al.'s study [10], the elastic modulus of two thermoformed aligners was measured at 2,427.9 ± 197.3 MPa and 1,958.5 ± 171.5 MPa. Consequently, a sample size of 5 was calculated for each group.

**Figure 1:**
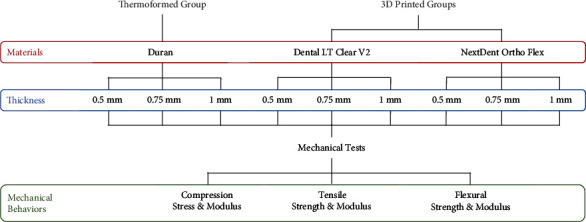
Experimental flowchart.

In this study, the materials were divided according to the method of retainer fabrication, which were thermoformed (Duran) and 3D-printed groups (Dental LT Clear V2, NextDent Ortho Flex). These specimens with different thicknesses (0.5, 0.75, and 1 mm) were used to evaluate compression, tensile, and three-point bending testing. The study design, depicted in Figure 1, involved preparing forty-five replicates for each material group. Each thickness comprised 15 replicates to conduct five replications per test. Consequently, a total of 135 samples were prepared according to the specified dimensions.

### 2.3. Preparing the Specimens

**Figure 2:**
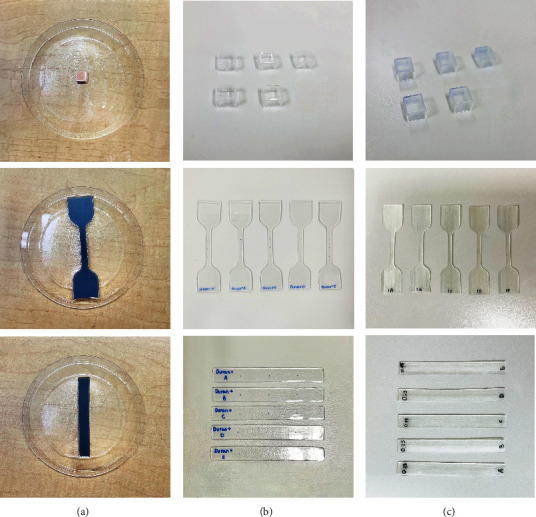
The first row (a) shows the model molds of the thermoformed group. The second row (b) displays the thermoformed specimens, and the third row (c) shows the 3D-printed specimens.

In the vacuum-formed thermoplastic group, the model molds were printed from the STL file by a stereolithographic (SLA) printer (Form 3B, Formlabs). The synthetic mold was positioned in a thermoforming machine caster (Biostar®; Scheu-Dental). The 0.5, 0.75, and 1 mm Duran thermoforming sheet (Scheu-Dental GmbH, Iserlohn, Germany) was adapted to the mold by the thermoforming process (Figure 2(a)). After that, molds were removed, and then, the specimen from each model was cut out and utilized for analysis (Figure 2(b)).

In 3D-printed groups, the specimens were designed by the Meshmixer program at different thicknesses of 0.5, 0.75, and 1 mm and then exported the STL file, and the manufacturing of samples was carried out with an SLA printer (Form 3B, Formlabs, Somerville, Massachusetts, USA) for Dental LT Clear V2 material (Formlabs Inc., Somerville, Massachusetts, USA). The specimens were first rinsed with isopropyl alcohol (IPA, ≥99%) for 15 minutes, then were subsequently positioned in a heated curing unit (Form Cure, Formlabs), and subjected to a 60-minute curing process at 60°C.

NextDent Ortho Flex material (Vertex-Dental B.V., Soesterberg, The Netherlands) was printed with a liquid crystal display (LCD) printer (Anycubic Photon Mono X). The samples were then submerged in two successive ultrasonic baths containing 95% ethanol, for a total of 5 minutes. Subsequently, the printed parts were transferred to a UV-light curing box (Form Cure, Formlabs) for final polymerization. Postcuring duration was 30 minutes, with an internal temperature of up to 80°C.

To diminish the impact of the environment, all specimens were stored in a consistent condition. The experiment had been performed at room temperature (23°C) and constant humidity (40–50%). In addition, before the mechanical testing, a digital micrometer screw gauge (Toolcraft, Georgensgmund, Germany) was used to measure each specimen's thickness.

The specimens are shown in Figures 2(b) and 2(c).

### 2.4. Compression Test

The hollow box-shaped specimens were selected to mimic the size of the first molar clinical crown for testing. These specimens are 10 × 11 × 7.5 mm in size with different thicknesses (0.5, 0.75, and 1 mm). The compression testing was done by applying a compressive force on each retainer specimen. The force was applied by the move of the upper plate at a rate of 1 mm/min under a 10,000 N load cell, while the lower plate stayed stationary. The universal testing machine (Instron Ltd., Buckinghamshire, England) was used for experiments.

The maximum load which the retainers can withstand, compressive stress, and compressive modulus were measured and recorded. The compression stress, *σ*, in megapascal (MPa) was calculated with the following formula:

(1) $$\begin{array}{c} \sigma =\frac{F}{A}, \end{array}$$

*σ* is the stress parameter, expressed in MPa; *F* is the force measured, expressed in Newtons; and *A* is the initial cross-sectional area of the specimen, expressed in square millimeters and the compressive modulus (E) in MPa with the formula:

(2) $$\begin{array}{c} \text{Ec}=\frac{\left(\sigma 2\;-\;\sigma 1\right)}{\left(\varepsilon 2\;-\;\varepsilon 1\right)} \end{array}$$

where Ec is the compressive modulus, expressed in MPa; *σ*1 is the stress, in MPa, measured at the value of the strain *ε*1; *σ*2 is the stress, in MPa, measured at the value of the strain *ε*2.

### 2.5. Tensile Test

**Figure 3:**
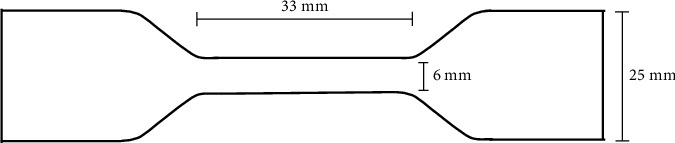
Tensile specimen type 5 (from ISO 527 part 3).

The tensile tests were performed according to the shape and size suggested by the normative EN ISO 527-3 (test conditions for films and sheets), type 5; however, they were conducted with different thicknesses (0.5, 0.75, and 1 mm). Detailed tensile specimens are illustrated in Figure 3.

To perform the tensile test, the adaptors were grips intended for tensile testing and an extensometer for strain measurement. The test was carried out at a rate of 5 mm/min under a 1,000 N load cell. Tensile strength and elastic modulus (MPa) were characterized and recorded. Experiments were conducted using the universal testing machine (Instron Ltd., Buckinghamshire, England) in accordance with the applicable ISO 527-3:2018 standard [11].

### 2.6. Flexural Test

In the three-point bending test, a strip of 80 mm length and 10 mm width was performed according to the standard ISO 178 (Plastics—determination of flexural properties). However, the samples were fabricated with different thicknesses (0.5, 0.75, and 1 mm).

The specimen was placed on two supporting pins, with a fixed distance apart on each thickness group. In this testing, the span was adjusted to span (L): thickness (h) ratio = 32 : 1. Span length for three-point bending test is described in Tables 2.

A three-point bending test was carried out (Instron Ltd., Buckinghamshire, England) with a constant displacement rate of 1 mm/min under a 100 N load cell. The maximum flexural strength (*σ*) and the flexural modulus (E) in MPa were calculated. Experiments were performed using the universal testing machine (Instron Ltd., Buckinghamshire, England) in accordance with the appropriate ISO 527-3:2018 standard [12].

### 2.7. Statistical Analysis

The normality of data was analyzed using the Kolmogorov–Smirnov test, demonstrating normal distribution. Data were descriptively analyzed using mean and standard deviation. The compressive stress, compressive modulus, tensile strength, elastic modulus, flexural strength, and flexural modulus were analyzed with two-way ANOVA with post hoc analysis using the Bonferroni test. The level of significance was set at 0.05. SPSS (version 23) was used for statistical analysis.

## 3. Results

### 3.1. Thickness

After thermoforming, the Duran sheet thickness was reduced. The thicknesses of the Duran specimens were 0.47 ± 0.03, 0.72 ± 0.03, and 0.95 ± 0.02 mm (4–6% lower than original sheet), while the 3D-printed group has a slightly greater thickness than the digital design. The thicknesses of Dental LT Clear V2 specimens in each group were 0.53 ± 0.02 mm, 0.77 ± 0.02 mm, and 1.03 ± 0.03 mm (2.67–6% greater than original digital design). The thicknesses of NextDent Ortho Flex specimens were 0.54 ± 0.04 mm, 0.79 ± 0.02 mm, and 1.04 ± 0.04 mm (5.33–8% greater than original digital design).

### 3.2. Compression Test

Two-way ANOVA showed that the compressive stress and modulus were significantly affected by thickness and the types of materials (*p* ≤ 0.001). The interaction between two factors (thickness and the types of materials) was also statistically significant (*p* ≤ 0.001). An increase in the maximum load, compressive stress, and compressive modulus tended to be proportional to the thickness. The highest compressive stress was Dental LT Clear V2 at thickness of 1 mm (51.47 ± 0.35 MPa), while the highest compressive modulus was Dental LT Clear V2 at thickness of 0.75 mm (717.22 ± 10.59 MPa). In a comparison between material types, the Dental LT Clear V2 and NextDent Ortho Flex were significantly more resistant to compression when compared to Duran (*p* ≤ 0.001), except for the 0.5 mm of NextDent Ortho Flex. The maximum load, compressive stress, and compressive modulus are measured and presented in Table 3.

**Figure 4:**
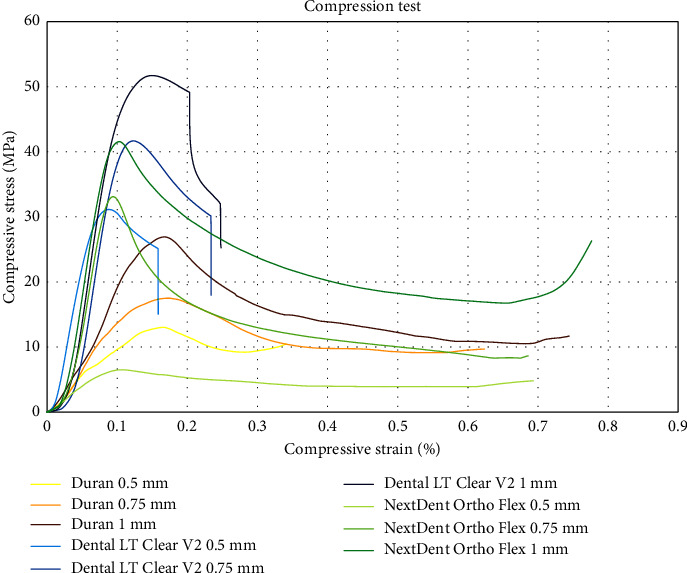
The stress-strain diagrams resulting from the compression tests conducted on Duran, Dental LT Clear V2, and NextDent Ortho Flex materials at thicknesses of 0.5, 0.75, and 1 mm.

When considering the stress-strain curves, the Dental LT Clear V2 groups, after reaching the elastic limit, would abruptly break apart into fragile fragments, while the other groups had an extended period of elongation until breakdown. The data are illustrated in Figure 4.

### 3.3. Tensile Test

The results of the two-way ANOVA indicated that the tensile strength and modulus values were significantly influenced by thickness and the types of materials (*p* ≤ 0.001). The interaction between two factors (thickness and the types of materials) was also statistically significant (*p* ≤ 0.001). Along with the increase in thickness of the materials, the tensile strength and elastic modulus tended to increase. Duran and NextDent Ortho Flex showed significant enhancement in these aspects as the thickness increased. On the contrary, there was no statistically significant difference in tensile strength between thicknesses for Dental LT Clear V2.

Among the types of material, Dental LT Clear V2 at all thicknesses demonstrated significantly higher tensile strength and modulus than both Duran and NextDent Ortho Flex (*p* ≤ 0.001). However, at the thickness of 1 mm, no significant difference in tensile strength was found between Dental LT Clear V2 and Duran. Tensile strength and modulus data are presented in Table 4.

**Figure 5:**
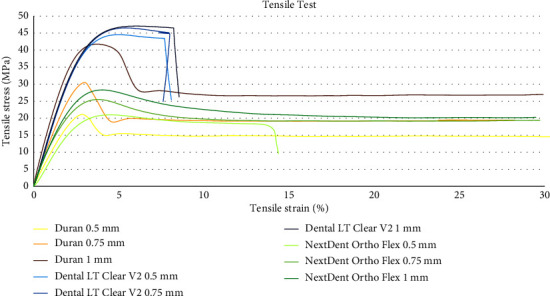
The stress-strain diagrams resulting from the tensile tests conducted on Duran, Dental LT Clear V2, and NextDent Ortho Flex materials at thicknesses of 0.5, 0.75, and 1 mm.

The stress-strain diagrams indicated that Duran exhibited the longest elongation, followed by NextDent Ortho Flex. The shortest elongation was present in specimens from Dental LT Clear V2.  Figure 5 illustrates the characteristic pattern of the tensile stress-strain curves.

### 3.4. Flexural Test

Two-way ANOVA revealed that the flexural strength and modulus were significantly affected by thickness and the types of materials (*p* ≤ 0.001). The interaction between two factors (thickness and the types of materials) was also statistically significant (*p* ≤ 0.001). Dental LT Clear V2 and Duran showed that decreased material thickness corresponds to increased flexural strength and modulus. However, there were no statistically significant differences in flexural modulus between Dental LT Clear V2 at a thickness of 0.5 and 0.75 mm. In contrast, NextDent Ortho Flex exhibited an inverse relationship, where greater thickness resulted in higher flexural strength and modulus. Nonetheless, among the three materials, NextDent Ortho Flex demonstrated the significantly lowest values for both flexural strength and flexural modulus (*p* ≤ 0.001). On the other hand, Duran exhibited the significantly highest flexural strength and modulus (*p* ≤ 0.001). Flexural strength and modulus data are presented in Table 5.

**Figure 6:**
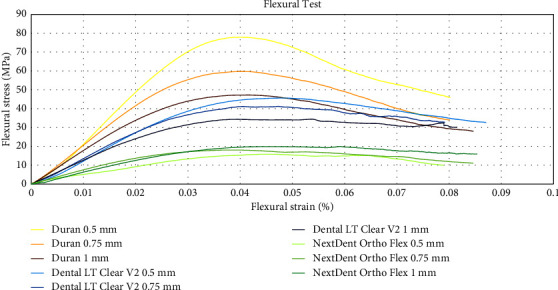
The stress-strain diagrams resulting from the flexural tests conducted on Duran, Dental LT Clear V2, and NextDent Ortho Flex materials at thicknesses of 0.5, 0.75, and 1 mm.

When compared among materials, Dental LT Clear V2 and NextDent Ortho Flex showed statistically lower flexural modulus and strength than Duran. The pattern of flexural stress-strain curves is shown in Figure 6.

## 4. Discussion

The implementation of direct 3D printing technology in customized dental appliances especially orthodontic retainers has become increasingly popular in clinical practice due to its strengths over the thermoforming method [13]. Dental materials utilized by companies to directly print retainers therefore are gradually increasing. However, available evidence regarding mechanical properties of direct printed clear retainers is limited. Despite a number of studies suggesting that different thicknesses of orthodontic appliances could affect their mechanical characteristics [10, 14], there is no consensus about the recommended thickness to be used. Therefore, the purpose of this study is to assess the mechanical behaviors of 3D-printed retainers in different thicknesses compared to thermoformed retainers.

The differences in thickness exerted an important influence on the compressive stress and modulus. The result of this study showed that with increase in thickness of specimens, higher compressive stress and modulus were observed. The highest strength and modulus were found in the specimens with the highest thickness (1 mm). In addition, the compressive resistance of the 3D-printed groups was higher than that of the conventional thermoform. The 3D-printed retainer can withstand a maximum load that was significantly higher than the thermoform Duran sheet, except for the NextDent Ortho Flex at a thickness of 0.5 mm. This finding is similar to the previous study by Jindal et al. [15] where they compared the compressive properties of 3D printing and thermoforming material. They concluded that the 3D-printed aligners could sustain mastication and biting force more reliably than thermoformed materials. Thus, 3D printable materials, such as Dental LT Clear V2 and NextDent Ortho Flex, may provide an ideal alternative material to conventional materials for dental retainers in terms of compressive resistance.

An essential mechanical property of the retainer is its resistance to compression forces. This characteristic is significant for assessing the mechanical strength of the retainer under compression forces equivalent to the bite force of humans [2]. Although the patient has been instructed not to wear the appliance while eating, there are still some unexpected forces such as clenching, grinding, or even the touch of the opposed teeth with unpredicted swallowing [16]. The biting force of healthy adult natural teeth in the molar region ranges from 210 to 527 Newtons (N) [17]. Thus, the compression resistance of the retainer should be at least 210 N. The majority of sample thicknesses evaluated in this study are capable of withstanding the general pressure of biting and mastication. However, the NextDent Ortho Flex with 0.5 mm thickness was deformed at 134.78 N before the minimum biting force was reached. Therefore, the 0.5 mm thickness of NextDent Ortho Flex may not be optimal for a retainer.

Elastic modulus and strength properties of the material are affected by its thickness [14]. This study confirmed that the alteration of specimen thickness had an influence on the average strength and modulus of elasticity. The tensile test of Dental LT Clear V2 showed that the increase in thickness of the printing retainers tended to result in an increase in the tensile strength and modulus, while the flexural test revealed an inverse relationship between the material elasticity and the sample thickness with the flexural strength and modulus being the lowest in samples of 1 mm thickness, and gradually increasing in the groups of 0.75 mm and 0.5 mm. This finding is consistent with the previous studies [10, 18, 19], which revealed that increased thickness leads to higher tensile strength and modulus but decreased flexural strength and modulus. It might be that thicker materials may exhibit heightened tensile strength due to an increased cross-sectional area; on the other hand, thinner specimens tend to exhibit greater flexural strength because of their influence on the rate of stress displacement when moving away from the material under tension [20]. However, NextDent Ortho Flex showed an increase in tensile and flexural modulus with the thickness. The variation in trends noticed in Dental LT Clear V2 and NextDent Ortho Flex could be a result of the printer's type (SLA or LCD) or the inherent properties of the materials involved [21, 22]. SLA printers exhibit reduced anisotropy in comparison with LCD printers due to the nature of the printing mechanism [22]. While SLA printers use a pinpoint laser for curing, LCD printers cure whole layers at once. Surprisingly, contrary to the assumption that SLA printers are less influenced by part thickness, our study's results suggest otherwise. This indicates that factors beyond layer-to-layer interactions alone play a role in determining the mechanical properties of 3D printing materials. For a more comprehensive understanding of the modes of failure, future studies could be done into a detailed analysis of fractured sites for each type of test using SEM imaging.

For the fabrication of the retainer, a thickness of each material with an appropriate modulus of elasticity is an important parameter to consider. The material needs to strike a balance, being sufficiently thick to withstand breakage and wear while maintaining a level of thinness that ensures comfort and tolerance. If a material showed an extremely high modulus, the retainer would be more rigid, and the patient would have difficulties inserting and removing the appliance. A material with low stiffness, on the other hand, will not produce sufficient force to retain teeth [23]. Consequently, most clear plastic retainers tend to be slightly thicker than aligners used in treating misalignments of teeth [6]. The most commonly used thicknesses of thermoformed retainers are 0.75 mm and 1.00 mm [24]. A previous study revealed that the fracture rate of clear retainers with 1 mm thickness was lower than that of those with 0.75 mm thickness [6]. However, the optimal thickness of 3D-printed retainer material remains uncertain. This study showed that Dental LT Clear V2 has significantly superior compression and tensile properties at all thicknesses compared to the thermoform group. When considering the patient's comfort and the desire to use a thinner thickness, it appears that a thickness of 0.75 mm for Dental LT Clear V2 provides sufficient properties. Although lower flexural strength and modulus were observed in Dental LT Clear V2, their values of 0.75 mm in Dental LT Clear V2 might be comparable to 1 mm in Duran. As a result, it can be concluded that 0.75 mm could be an acceptable thickness of Dental LT Clear V2 for retainer. Meanwhile, NextDent Ortho Flex groups showed the lowest mechanical properties at defined thicknesses for retainer when compared with the other materials. Therefore, this material may be suitable for producing intraoral devices with higher thicknesses than defined in this experiment.

The different types of retainer materials had a significant effect on the mechanical properties [25]. This study found that Dental LT Clear V2 outperformed Duran and NextDent Ortho Flex with identical thickness in terms of tensile strength and modulus, while Duran had significantly greater flexural strength and modulus. The variation in mechanical properties between materials could be attributed to variations in crystallinity, density, additives, degree of polymerization, and chemical composition. Most polymers are composed of crystalline and amorphous structures. Crystalline refers to the parallel structure of the polymer chains, whereas amorphous refers to their disorganized arrangement. The alterations in these structures of thermoplastic materials may be responsible for the increase in flexural rigidity and hardness [26]; meanwhile, the degree of polymerization of the 3D printing resin is strongly correlated with a greater elastic modulus and ultimate tensile stress [27]. Duran is made of PET-G, an amorphous and linear polymer comprising 1,4-cyclohexane, two methanol (CHDM), ethylene glycol (EG), and terephthalic acid (TPA) [28], which exhibits highly viscoelastic characteristics [29]. NextDent Ortho Flex contains 2-phenoxyethyl acrylate, which improves its flexibility; in contrast, Dental LT Clear V2 has the composition of bisphenol A and urethane dimethacrylate (UDMA), which contribute to its rigidity and strength. After the polymerization, the methacrylate monomer is converted into acrylic plastic, resulting in higher rigidity and brittleness of the material [30]. This may be a reason why the Duran and NextDent Ortho Flex showed a higher range and resilience in contrast to the Dental LT Clear V2 in the stress-strain diagram.

The limitation of the study is that the mechanical behaviors of material specimens were not evaluated in the intraoral simulation. Changes that occur intraorally during orthodontic retention may influence the mechanical efficiency of the retainers. Furthermore, as retainers are typically prescribed to be worn for extended periods of time, the durability of such materials and the financial implications of using different thicknesses are concerns for their cost-effectiveness. Unfortunately, this research is a cross-sectional study; therefore, this study might not present a complete reflection of clinical situations and the potential range of variations. Furthermore, research that mimics clinical conditions as well as cost-effectiveness for clinical use is necessary to fully comprehend the implications of the materials.

## 5. Conclusions

3D-printed retainers can achieve adequate mechanical properties when compared with thermoformed retainers. However, the thickness of the retainer can have a significant impact on its mechanical properties. Dental LT Clear V2 with a thickness of 0.75 mm achieved adequate mechanical properties, but further research is needed to investigate the appropriate thickness of NextDent Ortho Flex.

## Figures and Tables

**Table 1:** The composition of 3D-printed materials (their compositions were sourced from the material safety data sheets (MSDSs)).

Name of product	Composition
Duran® [8]	Polyethylene terephthalate-glycol copolyester (PET-G)

Dental LT Clear V2 [9]	Bisphenol A dimethacrylate
	Methacrylate monomer
	Photoinitiator
	Urethane dimethacrylate

NextDent Ortho Flex	2-Phenoxyethyl acrylate
	4-(1-oxo-2-propenyl)-morpholine
	Methacrylate ester monomer
	Diphenyl(2,4,6-trimethylbenzoyl) phosphine oxide
	Acrylate ester

**Table 2:** Span length for three-point bending test.

Span length, L (mm)	Thickness, h (mm)
16	0.5
24	0.75
32	1

**Table 3:** Mechanical properties from compression test.

	Material	Thickness (mm)		
		0.50	0.75	1.00
Load (N)	Duran	228.95 ± 27.11^A^	409.80 ± 30.38^B^	701.39 ± 18.31^C^
	Dental LT Clear V2	735.84 ± 13.20^A^	1,338.57 ± 28.81^B^	2,178.27 ± 15.97^C^
	NextDent Ortho Flex	134.78 ± 14.69^A^	1,012.88 ± 49.52^B^	1,838.87 ± 108.40^C^

Compressive stress (MPa)	Duran	14.05 ± 1.76^A^	16.10 ± 2.79^A^	25.10 ± 1.77^B^
	Dental LT Clear V2	31.53 ± 0.33^A^	41.87 ± 0.46^B^	51.47 ± 0.35^C^
	NextDent Ortho Flex	6.17 ± 0.37^A^	31.83 ± 1.44^B^	42.39 ± 1.28^C^

Modulus (MPa)	Duran	155.95 ± 53.87^A^	159.87 ± 28.04^A^	251.58 ± 41.72^B^
	Dental LT Clear V2	606.03 ± 13.74^A^	717.22 ± 10.59^B^	708.16 ± 45.11^B^
	NextDent Ortho Flex	122.30 ± 30.74^A^	624.98 ± 65.13^B^	715.45 ± 33.37^C^

This table presents the mean ± standard deviation of load, compressive stress, and modulus for each material at thicknesses of 0.5, 0.75, and 1 mm. Statistical analysis was conducted using a two-way ANOVA with post hoc analysis utilizing the Bonferroni test. The same capital letters in the horizontal raw mean there is no significant difference between materials at the 0.05% level. N, Newtons; MPa, megapascal; mm, millimeters.

**Table 4:** Mechanical properties from the tensile test.

	Material	Thickness (mm)		
		0.50	0.75	1.00
Tensile strength (MPa)	Duran	18.34 ± 4.04^A^	29.64 ± 1.13^B^	41.81 ± 3.69^C^
	Dental LT Clear V2	44.25 ± 0.91^A^	46.16 ± 0.57^A^	44.73 ± 2.55^A^
	NextDent Ortho Flex	19.18 ± 1.48^A^	22.36 ± 2.13^A^	27.24 ± 1.59^B^

Elastic modulus (MPa)	Duran	1,021.64 ± 47^A^	1,384.23 ± 69.58^B^	1,931.56 ± 52.51^C^
	Dental LT Clear V2	1,564.22 ± 139.72^A^	1,769.28 ± 138.64^B^	1,718.64 ± 60.83^B^
	NextDent Ortho Flex	871.41 ± 81.93^A^	1,177.48 ± 99.22^B^	1,359.13 ± 59.99^C^

This table presents the mean ± standard deviation of tensile strength and elastic modulus for each material at thicknesses of 0.5, 0.75, and 1 mm. Statistical analysis was conducted using a two-way ANOVA with post hoc analysis utilizing the Bonferroni test. The same capital letters in the horizontal raw mean there is no significant difference between materials at the 0.05% level. N, Newtons; MPa, megapascal; mm, millimeters.

**Table 5:** Mechanical properties from the flexural test.

	Material	Thickness (mm)		
		0.50	0.75	1.00
Flexural strength (MPa)	Duran	78.11 ± 0.76^A^	59.48 ± 1.50^B^	47.79 ± 1.54^C^
	Dental LT Clear V2	45.37 ± 0.98^A^	40.91 ± 1.83^B^	35.09 ± 3.99^C^
	NextDent Ortho Flex	16.26 ± 2.30^A^	17.30 ± 1.54^A,B^	20.28 ± 1.49^B^

Flexural modulus (MPa)	Duran	2,851.53 ± 148.41^A^	2,165.46 ± 87.46^B^	1,820.66 ± 140.41^C^
	Dental LT Clear V2	1,549.99 ± 57.35^A^	1,441.49 ± 65.76^A^	1,249.35 ± 119.35^B^
	NextDent Ortho Flex	597.54 ± 130.59^A^	660.66 ± 105.96^A^	747.81 ± 29.11^A^

This table presents the mean ± standard deviation of flexural strength and flexural modulus for each material at thicknesses of 0.5, 0.75, and 1 mm. Statistical analysis was conducted using a two-way ANOVA with post hoc analysis utilizing the Bonferroni test. The same capital letters in the horizontal raw mean there is no significant difference between materials at the 0.05% level. N, Newtons; MPa, megapascal; mm, millimeters.

## Data Availability

The data used to support this study are available from the corresponding author upon request.
